# Reducing Sweeping Frequencies in Microwave NDT Employing Machine Learning Feature Selection

**DOI:** 10.3390/s16040559

**Published:** 2016-04-19

**Authors:** Abdelniser Moomen, Abdulbaset Ali, Omar M. Ramahi

**Affiliations:** 1 Department of Computer Science, Rochester Institute of Technology, 1 Lomb Memorial Drive, Rochester, NY 14623, USA; axmvcs@rit.edu; 2 Department of Electrical and Computer Engineering, University of Waterloo, 200 University Ave W, Waterloo, ON N2L 3G1, Canada; abdulbasetali@gmail.com

**Keywords:** microwave sensors, nondestructive testing, feature selection, machine learning

## Abstract

Nondestructive Testing (NDT) assessment of materials’ health condition is useful for classifying healthy from unhealthy structures or detecting flaws in metallic or dielectric structures. Performing structural health testing for coated/uncoated metallic or dielectric materials with the same testing equipment requires a testing method that can work on metallics and dielectrics such as microwave testing. Reducing complexity and expenses associated with current diagnostic practices of microwave NDT of structural health requires an effective and intelligent approach based on feature selection and classification techniques of machine learning. Current microwave NDT methods in general based on measuring variation in the *S*-matrix over the entire operating frequency ranges of the sensors. For instance, assessing the health of metallic structures using a microwave sensor depends on the reflection or/and transmission coefficient measurements as a function of the sweeping frequencies of the operating band. The aim of this work is reducing sweeping frequencies using machine learning feature selection techniques. By treating sweeping frequencies as features, the number of top important features can be identified, then only the most influential features (frequencies) are considered when building the microwave NDT equipment. The proposed method of reducing sweeping frequencies was validated experimentally using a waveguide sensor and a metallic plate with different cracks. Among the investigated feature selection techniques are information gain, gain ratio, relief, chi-squared. The effectiveness of the selected features were validated through performance evaluations of various classification models; namely, Nearest Neighbor, Neural Networks, Random Forest, and Support Vector Machine. Results showed good crack classification accuracy rates after employing feature selection algorithms.

## 1. Introduction

Microwave Non-Destructive Testing (NDT) research is gaining increasing interest as its enables defects inspection of metallic surfaces and dielectric structures [1]. Different proposed microwave NDT sensors in earlier works operate over large frequency ranges with fine sweeping increments. For instance, metal defect sizing and detection under thick coating using microwaves from 8.2 GHz to 12.4 GHz was reported in [2]. In [3], a waveguide probe was used for crack detection in metallic surfaces with an operating frequency range of 12–18 GHz. In [4], detection of cracks in non-metallic materials using a microwave resonator was implemented by sweeping over a frequency range larger than 1 GHz. More recently, non-invasive measurements of complex permittivity based on sweeping the operating frequency of a microwave sensor from 1.7 to 2.7 GHz was reported in [5].

This work presents an implementation of machine learning feature selection methods to reduce the number of sweeping frequencies during microwave NDT. This, in turn, leads to a reduction in the detection circuit complexity and size of acquired data for reducing the costs of the testing equipment, especially for hand-held devices. A procedure for reducing sweeping frequencies in microwave NDT using machine learning feature selection is proposed in this paper. Employing feature selection methods to discover the most influential features among the full feature data set leads to minimizing the needed number of testing frequency points to perform NTD diagnostics. After selecting a subset of the important features, future structural health tests can be conducted using the selected features incorporated with supervised learning techniques for defect and damage detection using classification models. In fact, the idea of utilizing feature selection for minimizing sweeping frequencies is applicable for different NDT techniques.

As a validation study for this concept, a microwave waveguide-sensor loaded with split-ring resonators was used for detecting millimeter cracks in metallic surfaces as in [3,6]. The machine learning implementation in [6] was based on feature extraction using principal component analysis (PCA); however, in this study, the acquired data from the waveguide-sensor was processed using feature selection before applying machine learning classification to detect crack defects in the metallic surface under test.

From a machine learning perspective, feature selection is the process of identifying and removing as much irrelevant and redundant information as possible in order to reduce the dimensionality of the data and allow learning algorithms of the classification models to operate faster and more effectively. In other words, feature selection reduces the number of features and provides a better learning accuracy by selecting the important features from the original data set without mapping original data sets to a lower space [7,8]. Unlike feature selection, feature extraction methods such as PCA extracts the important features by mapping the original data set to a lower space. Features in the lower space do not correspond to the sweeping frequencies. The rest of the paper is organized as follows: Section 2 provides a description of the machine learning process and feature selection techniques. Section 3 describes the classification methods. The experimental setup and measured data set are described in Section 4. Section 5 presents the implantation and result analysis on the findings. Finally, Section 6 provides the conclusion of this study. The practical results show that the proposed approach can effectively assess the health of the metallic surface using two frequencies only.

## 2. Machine Leaning Background

Machine learning involves computational methods that improve the performance of mechanizing the acquisition of knowledge from experience [9]. It provides techniques for gaining insights into properties of data dependencies and significance of individual features in the data set. The methodology is based on feature selection and pattern classification for diagnosing the structural health. Feature selection techniques determine the important features to include in classification of a specific data collection [10,11]. Classification is a machine learning technique used to assign labels (classes) to unlabeled input instances based on discriminant features. In this work, as a validation study cracks in a surface of metallic structure were experimentally inspected. Class labels in this validation work are metallic structures’ status (healthy and unhealthy).

### 2.1. Machine Learning Process

**Figure 1:**
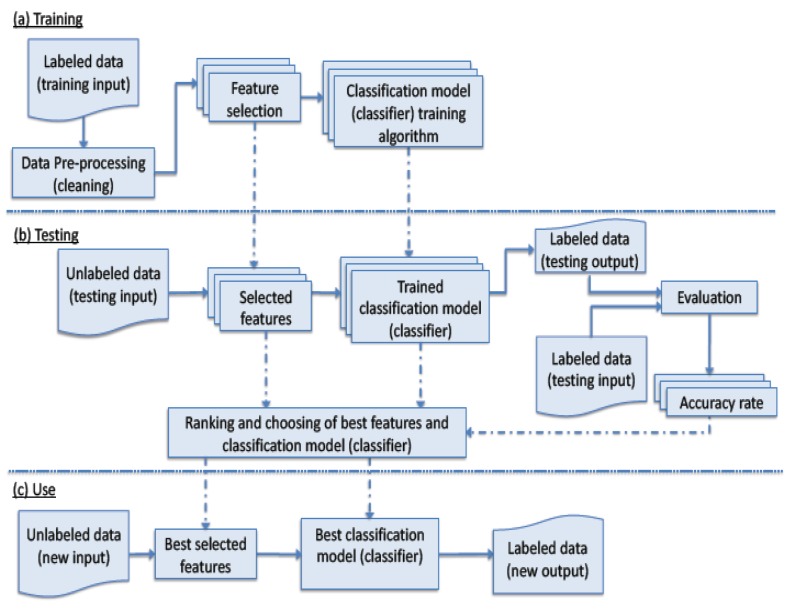
Machine learning process for classification.

The process of applying machine learning techniques to predict the class of unseen data is depicted in Figure 1. The process consists of three phases; (a) training (b) testing; and (c) use.

In the training phase, labeled data are collected; a subset of feature is selected, and then used to construct a classification model (classifier). The portion of the classified data that is used in the classifier training is referred to as the training data, and the part that are used to validate the classifier, the unseen data set, is referred as the testing data. Moreover, before training, data are pre-processed to be in proper format and free of anomalies such as missing outliers and erroneous data values, *etc*. There exist many feature selectors that generate different sets of features. Furthermore, there are various classifiers that can be trained to produce classification models [12].

When testing, the generated classification models from the training phase are used along with the chosen features. Reliable statistical evaluations are often utilized. For instance, classifiers are evaluated using part of the available data. These are unseen data that have known classes; hence, classifier accuracy could be calculated. Moreover, the size of the unseen data is determined in relation to the over-all size of the available data. Various measures are used. For a binary classification problem, F-measure is a widely used metric for evaluation and comparison of the results. F-measure is the harmonic mean of the precision and recall scores. Precision (P), recall (R) and f-measure (F1) calculations are shown in the following equations.

(1) $$P=\frac{TP}{TP+FP}\;R=\frac{TP}{TP+FN}\;F_{1}=2\frac{P\times R}{P+R}$$

P is the ratio of true positives (TP) from all predicted positive cases (true positive + false positive (FN) ). R is the percentage of true predict correct cases from the actual number of cases that should have been predicted as positive (TP+ FN). P, R, and F1 are useful measures when one of the classes is rare, *i.e.*, when the problem is an imbalanced classification problem. For multi-class classification problems, the overall classification accuracy measure is used and calculated as follows:

(2) $$AccuracyRate=\frac{\left(TP+TN\right)}{\left(TP+FN+TN+FN\right)}$$

In the use phase, the best classification model, according to the outcome of testing, is deployed, and when provided with values of the selected features of unseen instances, it determines their classes.

### 2.2. Feature Selection Techniques

Feature selection is the process of selecting a subset of relevant features for building learning models. When irrelevant features are eliminated from the original data set, the prediction accuracy of the models can be improved [11]. Quite often, data sets may contain features with different qualities, which can influence the performance of the entire learning framework. For instance, noisy features can decrease the classifiers performance. Moreover, some features can be redundant, and have high correlations. On the other hand, feature extraction is another technique for the feature reduction method that builds new features by extracting the most important information of the data set. Since the goal of this study to select the important frequencies (features) among the whole feature set, feature extraction methods are not applicable as they operate on all original features to build new projected features rather than selecting the important sub-features from the original features. Feature selection techniques can be classified into three categories: filters, wrappers, and embedded methods [13].

**Filter methods** select a subset of features as a pre-processing step, independently of the learning algorithms of the classifiers. They are based only on general features like the correlation with the variable to predict. Filter methods suppress the least interesting variables. These methods are particularly effective in computation time and robust to overfitting.

**Wrapper methods** utilize the classifiers performance to select feature subsets. They evaluate subsets of variables which allow detecting the possible interactions between variables.

**Embedded method**s perform feature selection in the process of training and are usually specific to given classifiers [14].

Filter-based feature selection techniques are adopted in this study. Four different filter-based feature selection and ranking techniques are investigated in order to identify the most important features; namely information gain, gain ration, relief, and chi-squared. The following subsections present an overview of these techniques.

#### 2.2.1. Information Gain (IG)

Information gain is the expected reduction in entropy obtained by partitioning the features according to a given feature. The entropy characterizes the uncertainty associated with a random collection of features, measuring the impurity or disorder of the data set. IG measures the amount of information in bits about the class prediction, if the only information available is the presence of a feature and the corresponding class distribution [15]. To select the important features from the data set, we calculate the entropy of the data set as a whole and for each class. For a given set of data D containing c different values (features), the entropy can be calculated as follows:

(3) $$Entropy\left(D\right)=-\sum_{i=1}^{c}P\left(c_{i}\right)\times \log_{2}\left(P(c_{i})\right)$$

where $P(c_{i})$ is the probability of getting the $i^{th}$ feature randomly selected from the data set D. If we make attribute $A_{i}$, with the v features, the root of the tree, this will partition data set D into v subsets D1,D2,... ,Dv. The expected entropy if $A_{i}$ is used:

(4) $$Entropy_{Ai}\left(D\right)=-\sum_{i=1}^{v}\frac{|D_{i}|}{|D|}\times Entropy\left(D_{i}\right)$$

Information gained by selecting attribute A to branch or to partition the data set is:

(5) $$Gain\left(D,A_{i}\right)=Entropy\left(D\right)-Entropy_{Ai}\left(D\right)$$

Equation (5) is used to select those features with the highest gain (discrimination).

#### 2.2.2. Gain Ratio (GR)

Gain ratio is a modification of the information gain method that prevents its bias; it is a normalized information gain. GR takes number and size of branches into account when choosing an feature. It overcomes the drawback of information gain by biasing the decision tree to rank the features of a high dimensional data sets. It corrects the information gain by taking the intrinsic information of a split into account. Intrinsic information is entropy of distribution of instances into branches (*i.e.*, how much info do we need to tell which branch an instance belongs to). Value of feature is decreased as intrinsic information gets larger [15].

#### 2.2.3. Relief

Relief is an instance-based algorithm that applies a ranking on features by allocating each a relevance weight [14,16]. The weight for a particular feature reflects its ability to distinguish the class values. Given enough data, the relief method has the potential to detect higher than pairwise feature interactions.

For each chosen instance, the nearest instance of the same class (nearest hit) and opposite class (nearest miss) are found. The feature’s weight is then updated according to how well its values distinguish the chosen instance from its nearest hit and nearest miss. It will receive a high weight if it differentiates between instances from different classes and has the same value for instances of the same class [15]. Relief randomly samples instances from the training data. Equation 6 shows the weight updating formula used by relief [17].

(6) $$W_{X}^{new}=W_{X}^{old}\frac{diff{\left(X,R,H\right)}^{2}}{m}+\frac{diff{\left(X,R,M\right)}^{2}}{m}$$

where $W_{X}$ is the weight for feature X, R is a randomly sampled instance, H is the nearest hit, M is the nearest miss, and *m* is the number of randomly sampled instances. The function diff calculates the difference between two instances for a given feature. For nominal attributes, it is defined as either 1 (the value of the attribute differs between the two instances) or 0 (the attribute has the same value in both instances), while for continuous attributes the difference is the actual difference normalized to the interval [0;1]. Dividing by *m* guarantees that all weights are in the interval [1,1] [17]. ReliefF is an extension of the original relief algorithm, it adds the ability to process multi-class problem as well as the ability of dealing with incomplete and noisy data. ReliefF method has the additional advantages of the applicability in the situations when data has low bias and has local dependencies, which other feature selection methods miss [14].

#### 2.2.4. Chi-Squared (Chi)

The chi-square distribution is one of the most widely used probability distributions methods for evaluating features individually [18]. It filters features similar to information-gain, gain-ratio and relief. The chi-square algorithm is based on the X2 statistics, and consists of two phases. The first phase begins with contentiously discretizing all numeric features starting with the significant level (sigLevel) until an inconsistency is exceeded in the discretized data. Each feature is sorted according to its values. Attributes discretized into one interval only will be removed. Phase 2 is a finer process of phase 1. Starting with significant level 0 determined in phase 1, each feature “i” is associated with the sigLevel[i], and takes turns for merging. Attribute “i” will not be involved in further merging if the inconsistency rate exceeded the sigLevel[i]. The process is continued until no feature’s values can be merged. At the end of this phase, if an feature is merged to only one value, it simply means that this feature is not relevant in representing the original data set. The feature selection is considered completed when the discretization ends [18]. The algorithm finds weights of discrete features basing on a chi-squared test [15].

## 3. Classifiers

The goal of classification is to accurately predict a target class for each case in a data collection. In this study, four classification algorithms were implemented to determine the sub-set of features that yields the highest accuracy. The classification techniques used in this study were the K-nearest neighbor, Random Forest, Neural Networks, and Support Vector Machine algorithms.

### 3.1. K-Nearest Neighbor

K-nearest neighbor algorithm (KNN) is part of supervised learning that has been used in many applications in the field of machine learning. The principle behind nearest neighbor methods is to find a predefined number of training samples closest in distance to the new point, and predict the label from them [15]. The nearest neighbor algorithm works in a similar decision tree algorithm in terms of classification, but, instead of finding a tree, you find a path around the graph or network and faster than decision trees. It classifies objects based on closest training examples in the feature space. KNN classification is based on an explicit similarity measure.

### 3.2. Neural Networks

Neural networks (NN) are bio-inspired algorithms for data processing to enable computers to learn similar to a human brain [17]. Neural networks are typically structured in layers which are made up of a number of interconnected nodes containing an activation function. Patterns are presented to the network via the input layer, which communicates to one or more hidden layers where the actual processing is done via a system of weighted connections. The hidden layers then link to an output layer where the outputs are presented. Most NNs contain a learning rule that modifies the weights of the connections [17].

#### Random Forest

The Random Forest (RF) is an approach that can also be thought of as a form of the nearest neighbor predictor. It starts with a standard machine learning decision trees [15,17]. Similar to single classification trees, RF grows many classification trees. It is essentially a meta estimator that fits a number of decision tree classifiers on various sub-samples of the data set and uses averaging to improve the predictive accuracy and control over-fitting [15].

### 3.3. Support Vector Machine

Support Vector Machine (SVM) represents a recent advancement in machine learning theory and delivers high performance in different applications. The SVM approach possesses many advantages. It is less computationally intense than other machine learning classification algorithms such as artificial neural networks. It performs well in high-dimensional spaces. It handles a nonlinear classification efficiently using the kernel trick that implicitly transforms the input space into another high dimensional feature space. SVMs belong to the general category of kernel methods [19,20], which are algorithms that depend on the data only through dot-products. Therefore, a kernel function can compute a dot product in some possibly high dimensional feature space. This has two advantages: first, the ability to generate non-linear decision boundaries using methods designed for linear classifiers. Second, the use of kernel functions allows the user to apply a classifier to data that have no obvious fixed dimensional vector space representation.

## 4. Experimental Setup

**Figure 2:**
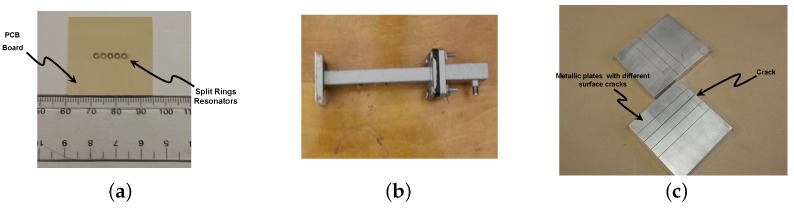
Photographs of the sensor. (**a**) used waveguide sensor side view; (**b**) printed circuit board (PCB) with split ring resonators; (**c**) a photograph of metallic plates with different cracks.

**Figure 3:**
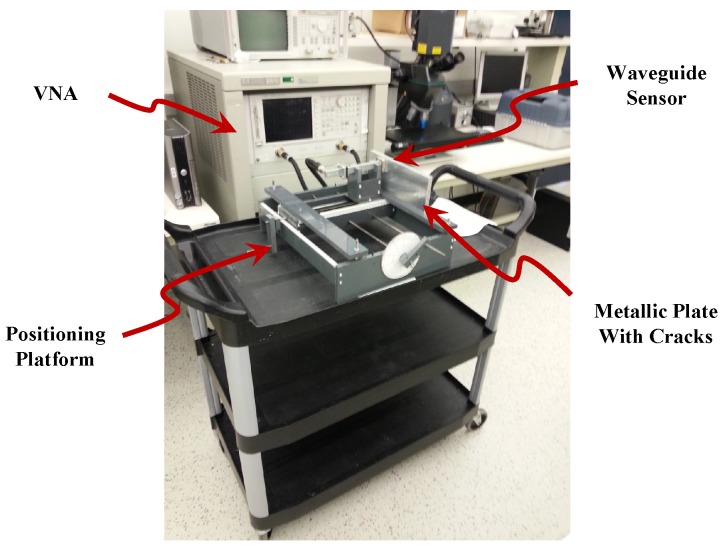
Testing configuration .

The measured data set was comprised of real measurements of metallic plate scans using a waveguide sensor loaded with meta-materiel particles [3]. The meta-materiel particles are arranged in an array as shown in Figure 2a. The waveguide was operated at the Ku-band and it has a cross section of 15.8 mm by 7.9 mm, with a standard flange with dimensions of 33.30 mm by 33.30 mm. Figure 2b,c show a photographs of the sensor and metallic plates with different clacks, respectively. The surface testing performed by scanning a metallic plate containing multiple 0.5 mm surface cracks ranging in depth from 0.5 mm to 2.25 mm. The sensor was connected to a vector network analyzer (VNA) in a one-port configuration as illustrated in Figure 3. The sensing mechanism consists of the VNA sending signals to the sensor at different frequencies in a sweep manner and collecting the reflected signals (data) from the sensor while the sensor scans the surface of the metallic plate under test at 0.5 mm stand-off distance. The VNA was swept over a frequency range of (12 to 18 GHz) with increments of 30 MHz to measure the reflection at 201 frequency points (features) for each scanned position.

### 4.1. Data Set Descriptions

**Figure 4:**
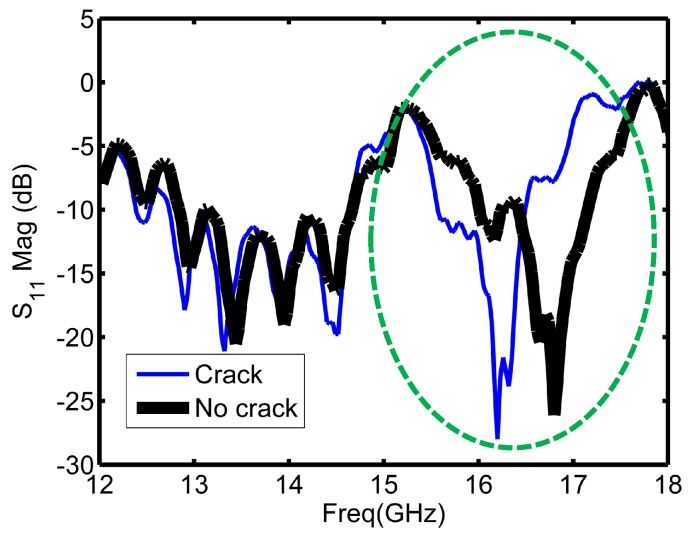
Reflection coefficient magnitude plots from healthy and unhealthy metallic surfaces.

Signals reflected back from the sensor contained information about the health of the scanned metallic plate. For example, the pattern of the reflected signal from a non-cracked (healthy) surface is different from the one reflected from a cracked (unhealthy) surface. Figure 4 illustrates the reflection coefficient magnitude over the operating frequency range for cracked and non-cracked surfaces.

**Figure 5:**
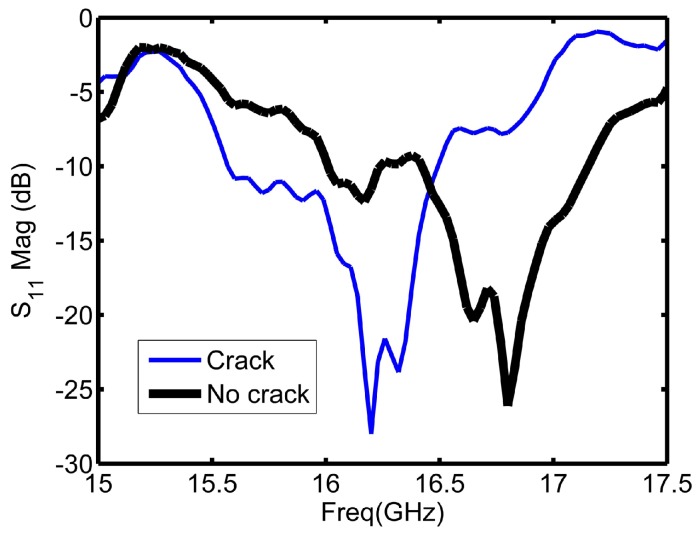
Reflection coefficient magnitude plot around the resonance frequency of the sensors.

As Figure 4 depicts, the sensor experienced a shift when encountered a crack as highlighted with the circle in Figure 4. The highlighted part of Figure 4 is the region around the resonance frequency of the sensor, and it is the main informative part in the plot about the structural health. Figure 5 shows a closer illustration of the reflection coefficient magnitude around the resonance frequency of the sensor. The range from 15.6 to 17.2 GHz is the range of interest for the coming implemented feature selection algorithms. In total, there were 53 frequency points (features) in this range starting from the 122nd feature to the 174th feature in the initial data set. The objective of the feature selection algorithms implemented in coming sections is reducing the number of sweeping frequency points by selecting the main informative frequency points among these 53 frequency points. Different classifiers were trained and tested for crack detection based on reduced data sets. The class distribution of the measured observations was symmetric (180 observations with a crack and 180 with no crack) to avoid unbalanced class scenario.

### 4.2. Pre-Processing

Input normalization data is very important when dealing with parameters of different units and scales. Therefore, all parameters should have the same scale for a fair comparison between them. In the absence of normalization, features with large values have a greater influence on the cost function when designing the classier. The data set understudy was normalized using min-max normalization which restricts the values of all features within predetermined ranges. The general formula is given as:

(7) $$x^{\prime }=\frac{x-min(x)}{max(x)-min(x)}*\left(b-a\right)+a$$

where x is the original value, $x^{\prime }$ is the normalized value, *a* and *b* are the min and max values of the new scale.

## 5. Results and Discussion

This section presents the outputs of the implemented feature selection techniques as well as their effect on the performance of the built classifiers.

### 5.1. Feature Selection Results

**Figure 6:**
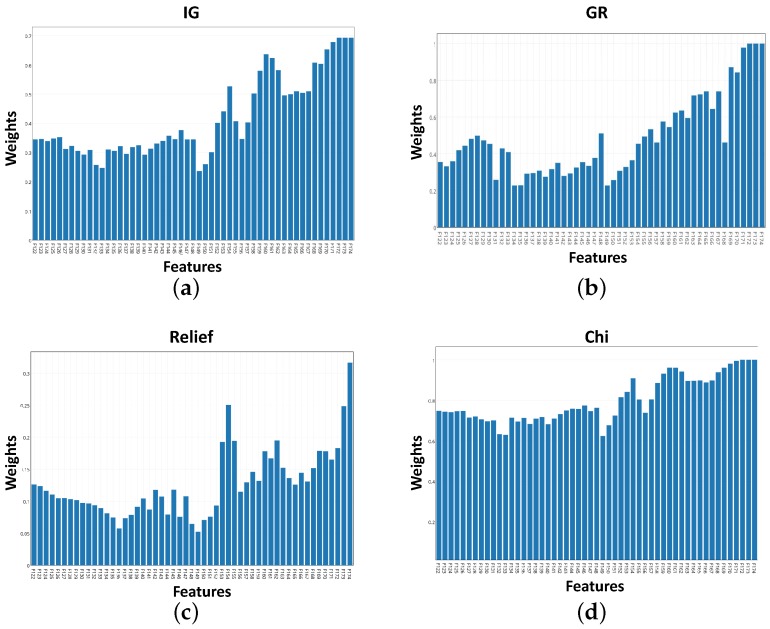
Weight plots of features for implemented algorithms. (**a**) weights *vs.* features using Information Gain; (**b**) weights *vs.* features using Gain Ratio; (**c**) weights *vs.* features using relief; (**d**) weights *vs.* features using chi.

In this study, information gain, gain ratio, relief,and chi-squared algorithms were implemented using the R-project [21] to select the top five important features among the set of 53 features in the vicinity of the sensor’s resonance. Weights of the features after applying the mentioned algorithms are shown in Figure 6a–d. In these plots, the higher the weight is, the higher the feature importance. As illustrated, weights using IG, GR and chi-squared algorithms have a common trend. However, relief algorithm selection was different compared to the rest of the algorithms.

Table 1 summarizes the outcomes of the feature selection algorithms. The top five features using IG and chi methods are identical. GR selection is similar to IG and chi with a difference only in the fifth feature. However, relief method selection was obviously different from the other implemented methods as depicted in Table 1.

### 5.2. Classification Implementation and Results

Deciding which classification algorithm to select in order to evaluate and classify the data set is one of the challenges in machine learning research. Predictive accuracy has often been used as one of the evaluation criteria for the predictive performance of classification or data mining algorithms. To overcome this issue, we have conducted experiments using different classification algorithms and used the classifier’s predictive accuracy on the experimental data set as the evaluation criteria.

Defect (crack) detection using reduced data sets has been evaluated using KNN, RF, NN, and SVM classifier models. The classifiers were tuned using grid search and cross-validation. Three levels of search grid where used for tuning parameters of implemented models. The SVM model was tuned in terms of the polynomial kernel degree and the regularization constant. NN models were tuned in term of number of hidden units and weight decay. Three odd levels (to avoid ties) of nearest neighbors were used for tuning KNN classifiers. The average classification accuracy was used as a criterion for model selection. In the view that the feature selection models returned the the top five features, five-dimensional data sets were used to build classifiers. Furthermore, an additional two-dimensional data sets were used for defect detection based on the first two important features. Results have revealed high classification accuracy rates. Table 2 and Table 3 summarize the five-dimensional and two-dimensional classification models, respectively. In total, 360 samples (observations) were used as follows: 270 observation for training using 10-fold cross validation and 90 observations held for unseen testing. More details about designing the training and test sets can be found in [22,23].

The average training accuracy rates were higher than 0.995 for all models. Furthermore, classification results indicate that the SVM outperformed the rest of the implemented models for all data sets as it scored 100% accuracy rate for all data sets. The strong performance of the SVM can be explained in view of the fact that the SVM measures the complexity of the hypotheses based on the margin with which it separates the data set, not the number of used features, which, in turn, leads to better generalization compared to other algorithms [24].

**Figure 7:**
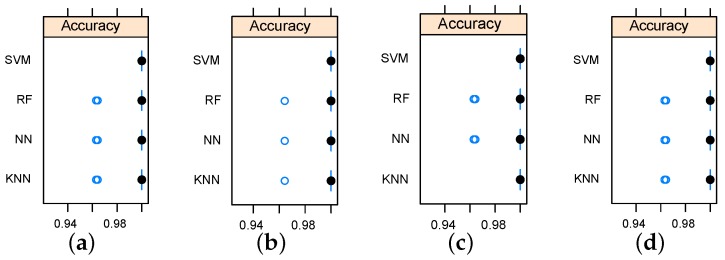
Box-and-whisker diagrams indicating the accuracy variation for the implemented classifiers. (**a**) based on the five top features using Information Gain (IG); (**b**) based on the five top features using Gain Ratio (GR); (**c**) based on the five top features using relief; (**d**) based on the five top features using chi-Squared.

**Figure 8:**
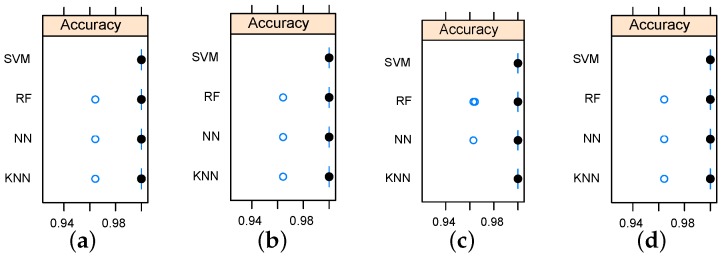
Box-and-whisker diagrams indicating the accuracy variation for the implemented classifiers using two-dimensional data sets. (**a**) using IG based data set; (**b**) using GR based data set; (**c**) using relief based data set; (**d**) using chi-Squared based data set.

Considering the relationship between data sets and employed classification models, the data sets obtained by relief feature selection led to better accuracy as the KNN reached an accuracy rate of 100% only when it was working on data sets generated by the relief algorithm. Accuracy variation shown in Figure 7 and Figure 8 indicate that the metallic plate surface can be tested with only two frequencies and an accuracy rate of 100% is achievable using SVM.

**Figure 9:**
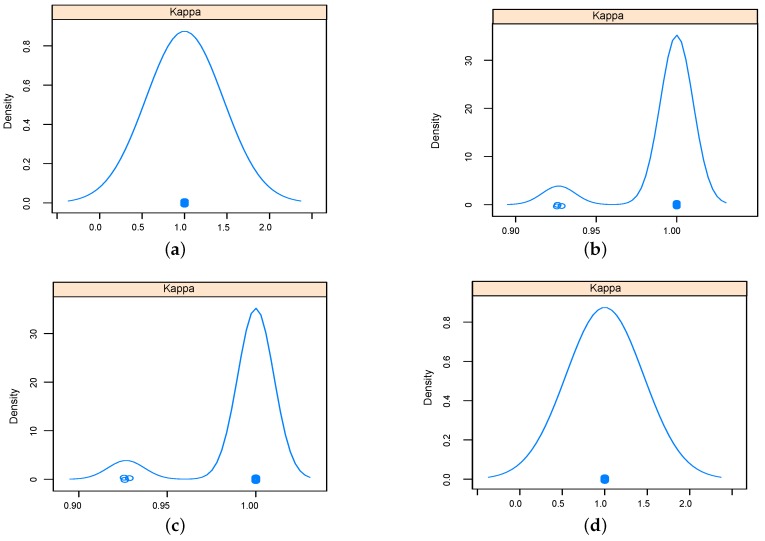
Kappa density plots for the implemented classifiers using five-dimensional data set using relief feature selection. (**a**) K-nearest neighbor (KNN); (**b**) Random Forest (RF); (**c**) Neural networks (NN); (**d**) Support Vector Machine (SVM).

**Figure 10:**
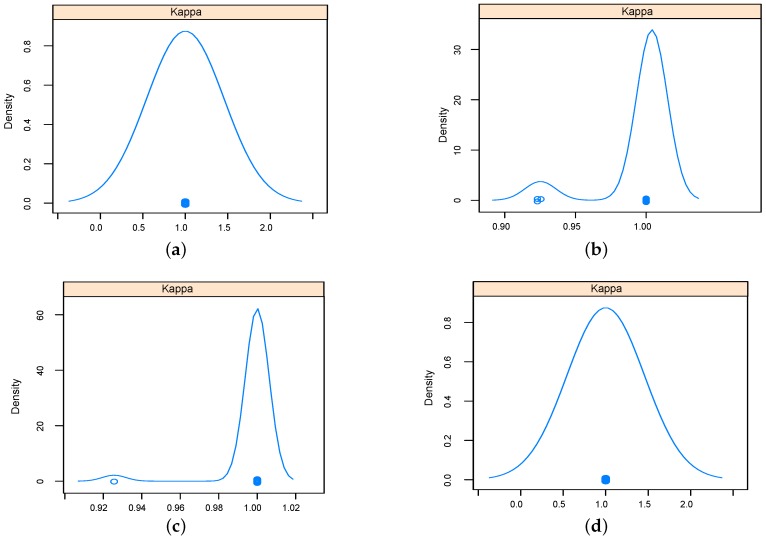
Kappa density plots for the implemented classifiers using two-dimensional data set using relief feature selection. (**a**) KNN (**b**) RF; (**c**) NN; (**d**) SVM.

The performance of implemented classifiers based on the data set obtained using relief feature selection technique was studied further in terms sensitivity and specificity, and results were reported as Kappa density [25] plots as in Figure 9 and Figure 10 for five-dimensional and two-dimensional data sets respectively. The kappa plots show that SVM and KNN outperformed RF and NN classifiers. Configurations of the classifiers operated on the data sets selected using relief feature selection method are listed in Table 4.

**Figure 11:**
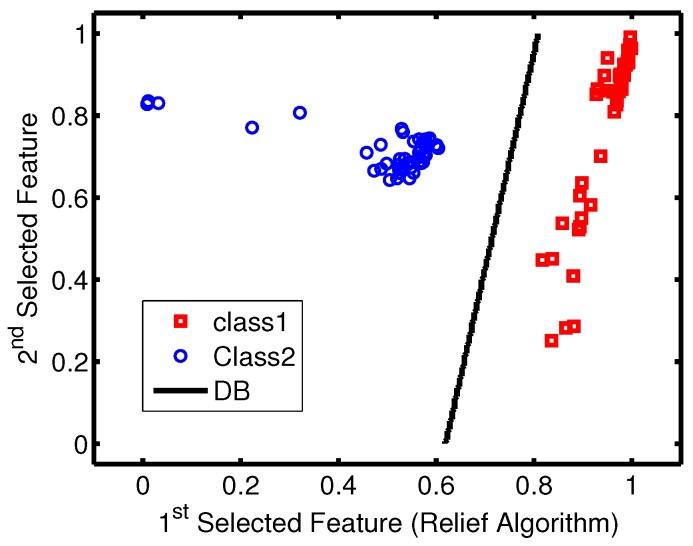
SVM decision boundary (DB) plot and unseen data from both classes.

Due to the strong performance in terms of the accuracy and kappa measures of the SVM classifier with all data sets, it has been selected as the final model among all implemented models. The two-dimensional SVM models have been tested using unseen data, and the learned decision boundary is plotted in Figure 11. As observed from Figure 11, the accuracy rate of the 100% was achieved.

## 6. Conclusions

This work demonstrated the employment of machine learning feature selection to reduce sweeping frequencies in NDT. Experimental measurements of metallic surface testing were reduced to five and two features only. Resultant data sets were evaluated using classification models including KNN, RF, NN and SVM. The accuracy rates for all implemented classifiers were higher than 0.995. Furthermore, based on the implemented classifiers’ performance, the relief selection algorithm was more effective than IG, GR and chi-Squared.

Considering the training and testing classification accuracy rates achieved by the SVM classifier (working on the two-dimensional data set selected using relief algorithm), the waveguide sensor can operate only at two frequencies achieving a classification accuracy rate of 100%.

Finally, we note that a major cost in developing frequency scanning detection systems is the frequency bandwidth. We emphasize that reducing the features to only two instead of many frequencies leads to significant reduction in the electronic circuitry for a real-world portable detection system and to a significant enhancement in time efficiency.

## Figures and Tables

**Table 1:** Top five important features using implemented feature selection algorithms.

Algorithm	1st Feature	2nd Feature	3rd Feature	4th Feature	5th Feature
IG	172	173	174	171	170
GR	172	173	174	171	169
Relief	174	154	173	155	153
Chi	172	173	174	171	170

**Table 2:** Classification average accuracy and standard deviation of 10 folds for K-nearest neighbor (KNN), Neural networks (NN), Random Forest (RF), and Support Vector Machine (SVM) classifiers using data sets of top 5-features of Information Gain (IG), Gain Ratio (GR), Relief, and chi-squared algorithms.

Model	Accuracy (Top 5 IG)	Accuracy (Top 5 GR)	Accuracy (Top 5 Relief)	Accuracy (Top 5 Chi)
KNN	0.9963 (1.16%)	0.9964 (1.13%)	1.00 (0%)	0.9963 (1.16%)
RF	0.9963 (1.16%)	0.9964 (1.13%)	0.9963 (1.16%)	0.9963 (1.16%)
NN	0.9976 (1.16%)	0.9964 (1.13%)	0.9976 (1.16%)	0.9976 (1.16%)
SVM	1.000 (0%)	1.00 (0%)	1.00 (0%)	1.00 (0%)

**Table 3:** Classification average accuracy and standard deviation of 10 folds for KNN, NN, RF, and SVM classifiers using first and second important features of IG, GR, Relief, and chi-squared algorithms.

Model	Accuracy (Top 2 IG)	Accuracy (Top 2 GR)	Accuracy (Top 2 Relief)	Accuracy (Top 2 Chi)
KNN	0.9964 (1.13%)	0.9964 (1.13%)	1.00 (0%)	0.9964 (1.13%)
RF	0.9964 (1.13%)	0.9964 (1.13%)	0.9963 (1.56%)	0.9964 (1.13%)
NN	0.9964 (1.13%)	0.9976 (1.13%)	0.9988 (1.17%)	0.9964 (1.13%)
SVM	1.000 (0%)	1.00 (0%)	1.00 (0%)	1.00 (0%)

**Table 4:** Configurations of the classifiers built using the data sets selected using relief feature selection.

	2-Dimensional Models	5-Dimensional Models
SVM	degree (1) regularization cost (0.25)	degree (3) regularization cost (0.5)
RF	variable per level (2)	variable per level (2)
NN	hidden units (3) weight decay (0)	hidden unit (5) weight decay ($10^{-4}$)
KNN	k (5)	k (9)
